# Measuring quality of life in patients with abdominal wall hernias: a systematic review of available tools

**DOI:** 10.1007/s10029-020-02210-w

**Published:** 2020-05-15

**Authors:** T. N. Grove, L. J. Muirhead, S. G. Parker, D. R. L. Brogden, S. C. Mills, C. Kontovounisios, A. C. J. Windsor, O. J. Warren

**Affiliations:** 1 Abdominal Wall Reconstruction Unit, Department of Surgery, Chelsea and Westminster Hospital, London, UK; 2 Department of Surgery and Cancer, Imperial College London, Chelsea and Westminster and the Royal Marsden Campus, London, UK; 3 Abdominal Wall Reconstruction Unit, Department of Surgery, University College Hospital, London, UK; 4 Department of Surgery, Royal Marsden Hospital, London, UK; 5 HCA Healthcare, London, UK

**Keywords:** Abdominal wall, Ventral hernia, Incisional hernia, Quality of life

## Abstract

**Introduction:**

Abdominal wall herniation (AWH) is an increasing problem for patients, surgeons, and healthcare providers. Surgical-site specific outcomes, such as infection, recurrence, and mesh explantation, are improving; however, successful repair still exposes the patient to what is often a complex major operation aimed at improving quality of life. Quality-of-life (QOL) outcomes, such as aesthetics, pain, and physical and emotional functioning, are less often and less well reported. We reviewed QOL tools currently available to evaluate their suitability.

**Methods:**

A systematic review of the literature in compliance with PRISMA guidelines was performed between 1st January 1990 and 1st May 2019. English language studies using validated quality-of-life assessment tool, whereby outcomes using this tool could be assessed were included.

**Results:**

Heterogeneity in the QOL tool used for reporting outcome was evident throughout the articles reviewed. AWH disease-specific tools, hernia-specific tools, and generic tools were used throughout the literature with no obviously preferred or dominant method identified.

**Conclusion:**

Despite increasing acknowledgement of the need to evaluate QOL in patients with AWH, no tool has become dominant in this field. Assessment, therefore, of the impact of certain interventions or techniques on quality of life remains difficult and will continue to do so until an adequate standardised outcome measurement tool is available.

**Electronic supplementary material:**

The online version of this article (10.1007/s10029-020-02210-w) contains supplementary material, which is available to authorized users.

## Introduction

Abdominal wall herniation (AWH) is an increasing problem for patients, surgeons, and healthcare providers. The incidence of ventral incisional hernia (IH) following major abdominal surgery is high, with rates of up to 20% following midline laparotomy [1, 2]. Advances in critical care and surgical technique are leading to improved survival following abdominal catastrophe in patients with multiple co-morbidities, with a resulting increasing burden of complex abdominal wall herniation (CAWH) [3]. Surgeons with a specialist interest in abdominal wall reconstruction within the context of a multi-disciplinary team (MDT), with careful recording of technique and outcomes [4, 5], are best suited to manage these complex patients.

Surgical-site infection, recurrence, length of stay, and mesh explantation are the most commonly utilised outcome measures in abdominal wall surgery, and these are improving, with many patients seeing excellent long-term results [6, 7]. However, a significant proportion of these operations are performed for symptom relief and to improve the quality of life (QOL). Despite this, QOL outcomes are recorded far less frequently than surgeon centred outcomes and surgical-site outcomes form the basis of the majority of publications from centres of excellence.

Assessment of QOL, both pre- and post-surgery, has been shown to be an important patient-reported outcome in all types of abdominal surgery [8, 9] and various tools are used. Generic QOL scoring systems have been used for many years, but disease-specific quality-of-life models are frequently more useful. A few data from specific AWH QOL-related studies exist [10, 11], and consequently, there remains an absence of consensus agreement surrounding the best way to measure QOL in AWH. Consistency in recording allows comparison between centres, techniques, and mesh type and position. The use of both generic and disease-specific QOL assessment tools is the current gold standard in surgery in general, and as such, a singly accepted tool for assessing QOL in AWH would appear to still be lacking. We hypothesise that there is no acceptable QOL assessment tool currently in use for patients undergoing abdominal wall hernia repair. The aim of this review is to systematically evaluate all currently available tools for measuring QOL (both generic and disease-specific) in this patient group to understand more thoroughly why this is the case.

## Methods

A focussed, systematic review of the literature was conducted by the first author (T.G.) under the guidance of a qualified medical librarian, in keeping with the Preferred Reporting Items for Systematic Review and Meta-Analysis (PRISMA) guidelines.

PubMed, MEDLINE, and EMBASE and Trip databases were used to search for comparative literature between 1st January 1990 and 1st May 2019. English language studies using quality-of-life assessment tools in abdominal wall hernias were included. The latest date for this search was 1st May 2019.

### Search string

1. To identify studies of AWH disease, we used the MeSH terms “abdominal wall hernia”, “incisional hernia”, “ventral hernia”, “recurrent hernia”, and “post-operative hernia”. There were combined with the keywords “abdominal wall reconstruction”.
2. To identify studies of quality-of-life assessment tools, we used the MeSH terms “Quality of Life”, “Patient recorded outcome(s)” combined with the key words “assessment”, “tool”, and “scale”.

A complete search string is shown in Table 1 in the supplementary material.

### Data extraction and review of studies

Articles were split into two groups and reviewers (TNG and LJM) independently extracted the following data points from the included studies: first author, year of publication, primary study centre, study design, type of hernia, quality of life tool, number of patients, percentage of patients with at least 1 year follow-up, type of hernia repair, mesh use, and timing of quality-of-life assessment.

### Inclusion criteria

Inclusion required the studies to utilise a formal quality-of-life assessment tool. Included studies must have reported on AWH, including IH and primary ventral hernias (VH). We defined a hernia as a musculofascial defect associated with protrusion of intra-abdominal viscera as described by the European hernia society [12]. This was further defined into primary or incisional and according to anatomical location. Quality-of-life assessment must be an identified outcome measure. Where reported, surgical site outcomes and complications were considered if part of the quality-of-life assessment. We stipulated no minimum follow-up time for inclusion.

### Exclusion criteria

Studies reporting groin hernias, lumbar hernias, and para-stomal hernias only were excluded. We excluded any study which reported on mixed hernia types if we were unable to independently extract the data pertaining specifically to AWH. Individual case reports and non-English language studies were excluded. We excluded paediatric studies (studies in patients under the age of 18) and studies focused on specific disease conditions (e.g., colorectal surgery and liver transplantation) other than AWH or VH.

### Citation management and screening

**Fig. 1:**
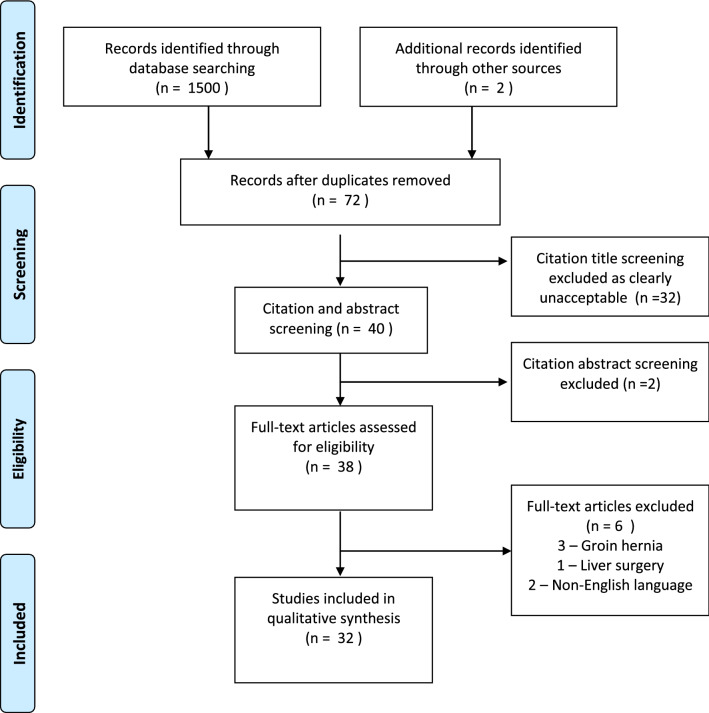
PRISMA diagram showing selection of studies for review

Initial search of the relevant databases identified a total of 1502 studies using the keywords outlined. After removal of duplicates and review of the title and the abstract, 95% of articles were excluded. All articles considered unsuitable for review were excluded (e.g., subject not AWH or QOL). Further review of the abstract and application of inclusion and exclusion criteria left 38 papers included for full review. Full article review leads to another 6 papers being excluded (3 groin hernia studies, 2 primary non-English language studies, and 1 study reviewing quality-of-life post-transplantation). A log of the matching process can be seen in the PRISMA flow diagram (see Fig. 1). Mendeley reference manager was used to manage citations (Mendeley Desktop v 1.19.4, UK).

### Data management and outcomes

Our primary outcome of interest is QOL assessment method in AWH patients. This will allow us to evaluate the different QOL assessment tools and better understand the current lack of consensus within this field. We extracted the timing of use of QOL assessment tool (preoperatively and postoperatively) for comparison of scores.

To assess participant characteristics, we aimed to identify if basic patient demographics were reported including sex, age, BMI, smoking status, and assessment of co-morbidities. If it was a comparative study, we aimed to identify if intervention and control group participant demographics were comparable. We also aimed to identify hernia size, mesh usage, mesh type, mesh placement, method of securing mesh, and adverse surgical outcomes.

Where there was conflict or disagreement between reviewers, this was resolved in a face-to-face meeting. Identified citations and outcome parameters were entered into a spreadsheet using Microsoft Excel for Mac 2011 (version 14.5.2).

## Results

The final review included 32 articles from an initial 1502 search results. The aggregated data from all 32 articles can be seen in Table 2 in the supplementary material.

### Case-mix and demographics

Thirty-two articles included a total of 6578 patients with AWH including VH, IH, recurrent ventral (RVH) and recurrent incisional hernia (RIH), across 25 sites in 9 countries. QOL was measured in all of the studies using a formalised assessment tool and was the primary outcome measure in 20 of the 32 included. In nine articles, it was the sole outcome measure. Other outcome measures included, most commonly, surgical site occurrence, and physical function such as strength and flexion. Type of hernia repair varied greatly across the studies and included open repair with and without mesh, laparoscopic repair with and without mesh, and anterior or posterior component separation. Twenty-two papers comment specifically on mesh being used for repair re-enforcement. Hernia size was referenced in 27 of the included studies, although a few studies report on the impact of hernia size on QOL. All studies included in the review assessed both male and female patients.

## Quality-of-life assessment tools

We found heterogeneity in the tools for assessment of QOL throughout the included articles. Seven used an abdominal wall-specific QOL tool, 11 used a hernia-specific QOL tool, 16 used a generic validated QOL tool, and 6 used a generic non-validated or independently devised QOL tool. Tool usage varied throughout the studies (see Table 1).

**Table 1:** QOL assessment tool use

QOL tool	Abdominal wall hernia-specific tool	Hernia-specific tool	Generic tool
SF-36	–	–	X
SF-12	–	–	X
WHO-QOL BREF	–	–	X
Eura-QuOL-5D	–	–	X
VAS	–	–	X
KPS	–	–	X
Analogue self-assessment scale	–	–	X
NS	–	–	X
PTSD PCL-5	–	–	X
CCS	–	X	–
AAS	X	–	–
HerQLes	X	–	–
References	[2, 9, 11, 13–27]		[28–40, 42]

*VAS* Visual acuity scale, *KPS* Karnofsky performance status, *NS* numeric scale, *CCS* Carolinas Comfort Scale, *AAS* Activities Assessment Scale, *HerQLes* Hernia-Related Quality of Life Scale

### Generic tools

SF-36 provides a QOL score based on mental and physical health and was the most commonly used QOL assessment tool seen in this review (12 studies of 1214 patients). However, only 2 [21, 29] articles used it as their sole method for collecting QOL data. Both saw improvement in mental and physical scores post AWR. SF-36 was used in combination with hernia-specific tools [25–27], AWH-specific tools [41], and other generic tools [22, 30–33]. Co-morbidities, BMI, and smoking status were reported separately.

SF-12 is a truncated version of SF-36 shown to give comparable results [50]_._ However, it was only used in two included studies [34, 35] (139 patients) for assessing post-operative QOL in AWH repair. Scott-Roth et al. [34] found that morbidity from biological mesh was higher than expected, but SF-12 QOL scores at 12 months were above baseline with 88% follow-up at 12 months*.* Rosen et al. [35] reported improvements in QOL using SF-12, in combination with an EQ-5D and EQ-VAS, following AWR with biosynthetic mesh in both the mental and physical domains.

EQ-5D is a standardised measurement of health-related QOL consisting of a questionnaire and a visual acuity scale EQ-VAS. It was designed to quantify generic patient-reported outcomes based on mobility, self-care, usual activities, pain/discomfort, and anxiety/depression. QOL scores from EQ-5D have been shown to be comparable to those using a hernia-specific tool (Carolinas Comfort Scale) following component separation (CS) mesh repair [22]. However, only 2 studies [22, 35] included in this review used EQ-5D and EQ-VAS, and although follow-up was good at 1 year (84–100%), the studies only included 138 patients. Furthermore, both used at least one other QOL assessment tool including SF-12 and the Carolinas Comfort Scale (CCS).

Karnofsky performance status (KPS) measures functional impairment due to any disease. Only one study [28] using KPS (in conjunction with SF-36) post-operatively in 71 patients at 1 year found that patients were limited to basic self-care and the inability to return to work was identified using this tool. KPS is not comparable to SF-36 and no pre-operative QOL scores were assessed using this tool.

WHOQOL-BREF provides a short-form QOL assessment in physical health, psychological impact, social relationships, and environment. It was used as the sole method for QOL assessment in one included study in this review [29] of 90 patients undergoing laparoscopic AWR with mesh. No significant improvement in quality of life after surgery was seen. Gender, BMI, smoking status, and time to return to work are not included in the 26-question tool, but are commented on separately by the authors.

### Hernia-specific tools

The Carolinas Comfort Scale (CCS) assess QOL based on movement, daily functioning, mesh sensation, and pain across a 6-point scale. Validated in all hernia types undergoing mesh repair; it is the only tool of its type. It was used in 11 studies including 4152 patients. It is the only tool of its type, and although it has been shown to be more sensitive for assessing QOL in hernia than generic tools [9, 10], it was used as the sole QOL assessment tool in only seven articles reviewed (see Table 2).

**Table 2:** Use of CCS QOL tool

References	CCS used as sole QOL tool	CCS used in combination with generic QOL tool
Blair et al. [2]	X	–
Colavita et al. [9]	X	–
Heniford et al. [11]	X	–
Ross et al. [18]	X	–
Kilma et al. [19]	X	–
Groene et al. [20]	X	–
De Paep et al. [22]	–	X
Jensen et al. [25]	–	X
Nielsen et al. [26]	–	X
Hope et al. [27]	–	X
Groene et al. [42]	X	–

Heniford et al. [10] assessed QOL using CCS in 3877 hernia patients undergoing repair with mesh in a multinational prospective study; one-third had AWH. With 87% follow-up at 1 year, improvements in QOL were seen across all domains apart from mesh sensation. The study’s ability to compare the relative merits of CCS compared to other tools was limited, as the cohort registry did not complete a generic QOL assessment.

Three articles [25–27] used SF-36 and CCS, albeit in only 174 patients. However, all showed correlation in comparative scores in improvement of QOL post-operatively between CCS and SF-36. Nielsen et al. [26] state 79% of patients preferred CCS to SF-36 and 83% considered it a better reflection of their QOL post hernia repair. Hope et al. [27] corroborate this reporting patient preference of CCS in favour of SF-36 at 3:1 in 56 patients. Body mass index, smoking status, and other co-morbidities do not feature in the tool, but were considered important to report by all of the studies.

### Abdominal wall hernia-specific tools

The Hernia-related QOL Survey (HerQLes) tool was designed in 2012 to assess QOL specifically in AWH [16]. The 12-point survey allows patients to score their own physical and emotional functioning. HerQLes was the most widely used AWH QOL tool in this review albeit in only five studies. QOL using HerQLes was reported on in 493 patients who underwent AWR with mesh highlighting AWH as a significant barrier to QOL. Feng et al. [15] showed that early hernia repair improves quality of life in cancer survivorship using HerQLes. Krpata et al. [13] also demonstrated improved post-operative QOL scores using HerQLes in AWH. Importantly, four of the five studies used HerQLes as their sole method of QOL assessment. All five studies made separate comments to co-morbidities, age, gender, and smoking status.

The modified activities’ assessment scale (AAS) assesses patient functioning and QOL [41] through questions surveying mood, lifestyle, and physical activity [43]. It was only used for QOL assessment in two of the included studies, both in the last 5 years. Cherla et al. [17] assessed QOL using AAS in patients with AHW undergoing reconstruction.

Post-operatively patients undergoing AWR experienced improved QOL. Some achieving QOL scores similar to the control, no hernia, group on the AAS. However, no use of a generic tool makes interpretation difficult. Langbach et al. [24] used AAS as well as SF-36 to assess QOL in AWH patients undergoing AWR reporting QOL scores using both tools were improved and comparable following mesh repair. Again, body mass index, age, and co-morbidities were matched across the groups and reported separately.

### Utilisation of QOL assessment

Fourteen studies included in our review sought to determine whether simply reconstructing an abdominal wall/repairing a hernia would improve QOL using a wide range of QOL tools. Despite this, these 14 studies failed to incorporate the full range of tools available. The remainder of the studies sought to determine the impact of certain pre-and peri-operative variables on hernia repair success and QOL, with a perceived inherent assumption that it would be increased by virtue of surgery. There was again a failure to utilise the range of currently available tools (see Table 3). QOL tools were used alone and in combination with other tools without dominance in one tool or one combination of tools being used within the field.

**Table 3:** QOL tool utilisation and rationale for study

Rationale for study	Repair of AWH on QOL	Mesh comparison in AWH repair	Tack comparison in AWH repair	Open vs laparoscopic repair of AWH	Mesh placement in AWH	Mesh usage in AWH	CS vs standard repair for AWH	Operative complication on QOL	Mental health impact of AWH
*QOL tool*									
SF-36	X	X	X	X	–	–	X	X	–
SF-12	X	–	–	–	X	–	–	–	–
WHO-QOL BREF	–	–	X	–	–	–	–	–	–
Eura-QuOL-D	–	–	–	–	X	–	X	–	–
VAS	–	–	X	X	X	–	X	–	–
KPS	X	–	–	–	–	–	–	–	–
Analogue self-assessment scale	X	–	–	–	–	–	–	–	–
NS	X	–	–	–	–	X	–	–	–
PTSD PCL-5	–	–	–	–	–	–	–	X	X
CCS	X	X	–	X	X	–	–	X	–
AAS	X	–	–	–	–	–	–	–	–
HerQLes	X	–	–	–	–	–	–	–	X

*VAS* Visual acuity scale, *KPS* Karnofsky performance status, *NS* numeric scale, *CCS* Carolinas Comfort Scale, *AAS* activities assessment scale, *HerQLes* Hernia-Related Quality of Life Scale

### Tool acceptability, completion rates, and follow-up statistics

Patient participation and follow-up at 1 year were poor throughout the studies. Twenty studies (65%) reported follow-up to 1 year. Follow-up ranged from 3 months up to 14 years [33]. Post-operative follow-up was varied and inconsistent across all the included studies. One study collected pre-operative data only, 21 studies collected pre- and post-operative data and 10 studies collected post-operative data only (see Table 4)*.* The majority of studies (65%) included more women than men and the mean patient age (45.9–64) was above average childbearing age with women of childbearing age significantly underrepresented. Impact of size of hernia was difficult to determine as it does not feature in any QOL assessment tool and hernias of all sizes are included in data sets without discrimination. However, as Nissen et al. have reported, larger hernias are likely negatively impacts on QOL.

**Table 4:** Timing of data collection

References	Pre-operatively only	Pre- and post-operatively	Post-operatively only
Blair et al. [2]	–	X	–
Colavita et al. [9]	–	X	–
Heniford et al. [11]	–	X	–
Krpata et al. [13]	–	X	–
Nissen et al. [14]	–	–	X
Feng et al. [15]	–	X	–
Criss et al. [16]	–	X	–
Cherla et al. [17]	–	X	–
Ross et al. [18]	–	–	X
Kilma et al. [19]	–	X	–
Groene et al. [20]	–	X	–
Snyder et al. [21]	–	–	X
De Paep et al. [22]	–	X	–
Alkhatib et al. [23]	–	–	X
Langbach et al. [24]	–	–	X
Jensen et al. [25]	–	X	–
Nielsen et al. [26]	–	–	X
Hope et al. [27]	–	X	–
Poelman et al. [28]	–	–	X
Bansal et al. [29]	–	X	–
Eriksen et al. [30]	–	X	–
Rogmark et al. [31]	–	X	–
Van Ramshosrt et al. [32]	X	–	–
ZarZaur et al. [33]	–	–	X
Scott-Roth et al. [34]	–	X	–
Rosen et al. [35]	–	X	–
Aho et al. [36]	–	X	–
Thomsen et al. [37]	–	X	–
Juvany et al. [38]	–	–	X
Bansal et al. [39]	–	X	–
Asti et al. [40]	–	X	–
Groene et al. [42]	–	–	X

Comment on patient post-operative satisfaction was noted in a number of studies [28–31], but the report on satisfaction with the quality-of-life tool used was sparse. Patient preference for a hernia-specific tool over a generic tool was observed in two studies. Nielsen et al. [26] specifically reported on patient satisfaction and perceived acceptability of tools where 75% of patients included preferred CCS to SF-36 due to ease of understanding and 83% felt it to be more specific to their situation. Hope et al. [27] corroborated this. Patient satisfaction with AWH or preference.

## Discussion

Abdominal wall reconstruction as a specialty in its own right is growing rapidly. Unlike other specialities, it mostly consists of elective cases performed to improve quality of life. This allows for thorough pre-operative planning and patient discussion. A systematic review undertaken by Jensen et al. [44] reviewed the then available QOL assessment tools and determined none were universally accepted. Despite the recognised need for a standardised method for quality-of-life assessment in AWR still, no one tool has become dominant. More recently, Sando et al. in 2020 have published a comprehensive systematic review assessing long-term patient-recorded outcomes from ventral hernia repair with mesh specifically. They have shown that some factors used for assessing QOL may have improved following surgery. A wide range and varied use of QOL assessment tools were again noted and drawing strong conclusions is perhaps impeded by difficulties in comparing outcomes because of this. This review was performed to provide up-to-date information with the emphasis on determining the use and acceptability of currently available tools for the AWH population to understand why this is still the case.

We have identified significant heterogeneity within the AWH literature with a total of 14 different QOL tools being used across 32 studies. Furthermore, we have seen two distinct sub-groups of studies; the first are studies using QOL tools to assess if repairing AWHs improves QOL in patients at all. The second utilise QOL tools to measure any difference in outcome between different techniques or interventions. Here, mesh position and plane development were frequently reported in studies comparing operative technique, but analysis of any relationship to QOL is frustrated by the absence of a common nomenclature to describe mesh placement [45]. It is somewhat surprising that this group only includes 14 studies given that presumed improvement in QOL secondary to surgery is one of the key factors in patient and surgeon decision-making. Timing of data collection and follow-up duration varied greatly across the studies, suggesting that optimum follow-up time also remains unclear.

Generic QOL tools provide a general overview of perceived QOL, whereas disease-specific surveys are useful in detecting the change in health and QOL as the result of a specific condition and/or the impact of a given focused treatment [39]. They are long established and widely accepted in their role measuring all health-related QOL. SF-36 and SF-12 have been used in the literature since 1989 with similar results [46]. They are extensively validated and give a ‘common language’ to researchers. They are adaptable and translatable within the general populations. However, their ability to assess specific pathology populations limited [47]. This was further highlighted by the National Health Service in the UK as part of their national patient-recorded outcome measures’ (PROMs) programme. It found, following a survey of 69,677 patients with inguinal hernia in 2016/17, that generic or non-hernia-specific PROMs were of limited value, and consequently, inguinal hernia was removed from the programme. AWH patients have different experiences both pre- and post-operatively compared to the daily lives of the ‘normal population’ [48] and have often suffered a significant surgical history with subsequent psychological impact [23]. Consequently, we have found in the last decade, hernia-specific QOL tools have gained popularity. In particular, CCS has been validated in large cohorts and shown to give a better representation of patients’ quality of life following hernia repair with mesh [11] and is preferable with patients.

Generic tools are still the most commonly used QOL assessment tools within the AWH literature. Where hernia-specific tools are used, it is often in combination with a generic tool; perhaps, to ensure that some comparable data are collected using the more validated, established method. Perhaps also highlighting inadequacies within the available disease-specific tools. General hernia-specific tools may be more focused than generic tools and their use is well established within the groin hernia literature [9]. However, we are concerned that they do not contain some specific domains important in the AWH population. We are also concerned that filling in two questionnaires is time-consuming for patients and may lead to a reduction in engagement and potential misrepresentation of outcomes. This has led to the development of AWH QOL-specific tools. Availability and uptake of AWH-specific tools have also been fairly limited. The European Hernia Society has developed and published the Eura-HSQOL in 2016 designed for use specifically in patients undergoing AWR as it was felt that other tools for evaluating QOL after hernia repair have not proven useful. This would appear to confirm a reluctance to consistently use other available tools. Unfortunately, its uptake has also been limited and it was not used in any identified studies within our review. Why utilisation and uptake remain low is unclear and from our review further research appears still required for using an AWH-specific tool. We suspect that this may be because AWH-specific tools still omit certain factors which may be important in measuring quality of life such as age, sex, co-morbidities, and smoking status. Furthermore, to our knowledge, they have all been developed by surgeons without patient input.

Patient-specific factors, behaviours, and co-morbidities are established contributors to QOL outcomes in major abdominal surgery, such as colorectal resections [48]. In this review, the majority of studies (26 studies) reported patient-specific factors separately including sex, age, BMI, diabetes, smoking, cardio-respiratory function, steroid use, previous surgery, and tobacco smoking. The most commonly used generic QOL tools do not collect these data when assessing quality of life. Whilst these demographics are not quality-of-life measurements in themselves, they can negatively contribute to increased post-operative morbidity, increased length of hospital stay, and increased risk of hernia recurrence [49]. Recurrence significantly negatively affects QOL and can reduce it to below pre-operative baseline [15, 24, 39]. Such factors appear correlated to QOL and measuring them may be useful. The impact of hernia size on QOL was poorly reported. Where compared large hernia size was seen to negatively impact on QOL. However, the literature is heterogeneous and often patients with different size hernias are bundled together. Patients with different hernia sizes have different expectations and experiences, and therefore, how their hernia impacts their QOL will differ. Current AWH QOL tools do not take into account hernia size or hernia defect size meaning that we cannot discriminate when comparing the tools. This makes interpreting results difficult and is a limitation of the currently available tools. Patient satisfaction is not included in QOL assessment tools, but has been reported in a number of included studies. While measuring patient satisfaction as a proxy of QOL may not be ideal, a satisfied patient may have perceived improvement in QOL relative to a dissatisfied patient. How a patient feels about their surgery could also introduce bias into their responses when completing a QOL assessment and as such should perhaps be accounted for.

Ability to work, and time to return to work are an important outcome measure for many patients and wider society as a whole. Many AWH patients are of working age and there must be a negative economic impact on them being unable to do so. Although work activities are included as a score in the ASS; occupation, demographics, significant other diagnoses, impact on body image, and co-morbidities are not accounted for in the CCS, HerQLes, or short-form surveys. Gender impacts scores in the SF-36 and SF-12 QOL assessment methods with women regularly scoring lower than men [50], but its impact on disease-specific tools is unknown. There is limited evidence in the literature on abdominal wall repair in pregnancy and no consideration or assessment of pregnancy, fertility, or fecundity in hernia QOL tools.

Ultimately, assessing QOL is a patient-reported outcome and as such is subjective. Factors impacting most greatly on QOL are likely to vary from patient to patient. Including aspects important to patients, rather than clinicians, can only be achieved through patient participation and involvement in developing a universally accepted tool. For some patients, certain factors will hold more weight than others and pre-operative discussion and decision-making should be focussed towards an individual patient expectations. Decision-making in AWH surgery remains difficult. This said, the use of such a suitable and acceptable universal pre-operative QOL measurement and/or being able to predict subsequent QOL improvements in patients undergoing successful surgery would aid decision-making for the patient and the surgeon. QOL measurement can and should be a valuable adjunct in decision-making.

## Limitations

### Risk of bias

Existing reference tools were analysed and used to assess the methodology of the studies to determine if studies were at “low, “high”, or “unclear” risk of bias. The methodology of the studies was analysed the quality of the study which was evaluated using the Cochrane risk of bias tool for randomised-controlled studies and the ROBBINS-I tool for non-randomised or observational studies. However, this was not accounted for as part of the review as all studies that underwent full literature review and fulfilled the inclusion criteria were included.

### Exclusion of non-English language studies

English language studies were excluded from this review. While inclusion may have led to perceived inaccuracies in translation and interpretation due to language barriers, we accept that there may be relevant data overlooked.

## Conclusions

This review highlights significant variation in the adoption and utilisation of QOL tools in the AWH literature. No one tool has become dominant in this field. Hernia-specific QOL tools are becoming more popular. However, the most frequently used scale is still the generic SF-36 most likely due to perceived inadequacies in hernia-specific QOL tools or fears that without a well-known, validated, established tool being used the data will not be published. Regardless of which tool is used, there may be key aspects to being an AWH patient that are still not being captured by any of them.

Assessment, therefore, of the impact of repair, or different techniques, on quality of life remains difficult. More work is required to refine and develop these QOL tools to help guide management and to embed validated, structured QOL measurement into patient and surgeon peri-operative decision-making. A single, user friendly, tool incorporating components from disease-specific and generic QOL tools that is can be used for a standardised period of long-term follow-up may provide this.

## Electronic supplementary material

Below is the link to the electronic supplementary material.

Supplementary file1 (DOCX 40 kb)

Supplementary file2 (DOCX 259 kb)
