# Supplementary material for: Measuring quality of life in patients with abdominal wall hernias: a systematic review of available tools
Source: Hernia. 2020 May 15;25(2):491–500. doi: 10.1007/s10029-020-02210-w (PMC8055629; doi:10.1007/s10029-020-02210-w)
Supplement: Supplementary file 1 — Supplementary file1 (DOCX 40 kb) [file 10029_2020_2210_MOESM1_ESM.docx]

| **Group** | **Search Terms** |
| --- | --- |
| Abdominal Wall Hernias | “Abdominal wall” OR “Incisional” OR ‘Ventral” OR “Post operative” AND “Hernia” AND “Abdominal wall reconstruction” |
| Quality of Life | “Quality of Life” OR “Patient recorded outcome” OR (“Patient” AND “Reported” AND “Outcome” AND “tool” OR “scale” |
| Conjunctions | AND, OR |

*Table 1. Search String*
