# Supplementary material for: Measuring quality of life in patients with abdominal wall hernias: a systematic review of available tools
Source: Hernia. 2020 May 15;25(2):491–500. doi: 10.1007/s10029-020-02210-w (PMC8055629; doi:10.1007/s10029-020-02210-w)
Supplement: Supplementary file 2 — Supplementary file2 (DOCX 259 kb) [file 10029_2020_2210_MOESM2_ESM.docx]

| KEY |  |  |  |  |
| --- | --- | --- | --- | --- |
| AWH - Abdominal Wall hernia | VIH - Ventral Incisional Hernia | AWR - Abdominal Wall reconstruction | CS - Component Separation | - = not stated or incomplete data |
| VH - Ventral Hernia | IH - Incisional hernia | QuOL - Quality of Life |  |  |

*Table 2. Data extraction from included studies within the review with key.*
